# Mechanically-Induced Long-Period Fiber Gratings Using Laminated Plates

**DOI:** 10.3390/s20092582

**Published:** 2020-05-01

**Authors:** Ismael Torres-Gómez, Daniel E. Ceballos-Herrera, Karla M. Salas-Alcantara

**Affiliations:** 1 Centro de Investigaciones en Óptica AC, Loma del Bosque 115, León Gto. 37150, Mexico; alrak2212@gmail.com; 2 Instituto de Ingeniería, Universidad Nacional Autónoma de México (UNAM), Cd. Universitaria, Alcaldía Coyoacán 04510, Mexico; DCeballosH@iingen.unam.mx

**Keywords:** mechanically-induced long-period fiber gratings, leading rejection band, coarse wavelength division multiplexing

## Abstract

This work presents a formation method of mechanically-induced long-period fiber gratings using laminated plates. The mechanically-induced long-period fiber grating is temporarily inscribed by compressing the optical fiber between a flat plate and the proposed laminated plate. In turn, the new laminated plate consists of a parallel assembling of single-edged utility blades. We present the experimental characterization of mechanically-induced long-period fiber gratings while employing three laminated plates with a period of 480 ± 20 µm and low duty cycles. These mechanically-induced long-period fiber gratings display a leading rejection band (>15 dB) with a couple of shallow rejection bands (<2 dB) in the range of 1100–1700 nm. This spectral behavior is due to the new mechanical fabrication process that is based on laminated plates that we have proposed, which consists of piling multiple blades with trapezoidal edges that are polished with different levels to obtain different duty-cycles. With the proposed method, we can obtain values of duty-cycles around 10%, much lower than those obtained using traditional methods. Additionally, with this new method, the required mechanical pressure to form the grating is remarkably reduced, which minimizes the probability of the optical fiber failure in the mechanically-induced long-period fiber gratings (MI-LPFGs). Moreover, the proposed mechanically-induced long-period fiber gratings with a single rejection band open the feasibility to implement coarse wavelength division multiplexing systems that are based on long-period fiber gratings.

## 1. Introduction

Long-period fiber gratings (LPFGs) are wavelength-selective components that allow the energy coupling from the fundamental core mode to the co-propagating higher-order cladding modes. This modal coupling results in multi rejection bands in the optical transmission, in which their resonant central wavelengths depend on the phase-matching condition [1]. The rejection bands in LPFGs have found applications in telecommunications, sensing, and optical fiber lasers [2,3,4]. Since the end of the 90s, to date, many techniques have been reported in the LPFGs inscription, such as UV, CO_2_, femtosecond irradiation, electric arc, and mechanically-induced between other methods [1,5,6,7,8]. Today, most of these techniques have been improved to achieve LPFGs with specific physical properties for different applications [9,10,11,12]. Recently, other methods have been proposed using the attractive LPFGs inscription process [13,14,15]. 

Mechanically-induced long-period fiber gratings (MI-LPFGs) are a particular case of LPFGs that was proposed by S. Savin et al. in 2001 [8]. The MI-LPFGs are implemented by a periodical index modulation in the core and the cladding along the optical fiber by applying mechanical pressure, microbending, or a combination of both. As it is well known, the MI-LPFGs offer similar spectrum transmission properties with respect to LPFGs that are inscribed by the other methods. However, MI-LPFGs stand out because they can be implemented in any single-mode fiber at a low-cost production. Additionally, MI-LPFGs do not require a stability process after being implemented, as compared to other methods. Additionally, MI-LPFGs are wide tunable with flexible control of depth attenuation and bandwidth of the rejection bands, and they are reversible. These properties make MI-LPFGs attractive for actual uses, such as variable gain shapers, mode converters, wavelength filters, and sensors, among other applications [16,17,18,19,20,21,22,23,24,25,26]. 

The methods commonly employed to MI-LPFGs include; grooved plates, winding strings, coil springs, screws, and even acoustic waves [5,27,28,29,30]. Among this variety of options, the grooved plate is the most widely used method, because it has a simple fabrication and easy implementation. However, the MI-LPFGs by grooved plates present some constraints. First, the period size in the grooved plate is limited by the machining process to be less than 450 µm. This period size restricts the mode coupling between the fundamental mode in the core and high order modes in the cladding in the range of 1200–1700 nm. As is well known, the mode coupling to high order modes in cladding is desirable because their rejection bands present higher sensitivity to perturbations from the surrounding environment. In the same way, this size of period hinders achieving rejection bands with great attenuation depth below 1.4 µm in single-mode standard optical fibers. On the other hand, the machining process stunts to have a duty cycle lower than 0.5 for the above mentioned period length. As expected, a large duty cycle entails higher uniaxial pressure, and this leads to a higher probability of failure in the optical fiber. In this sense, it is a challenge to explore new plate configurations to overcome these drawbacks or propose MI-LPFGs with new spectral transmission features.

In this work, we propose and experimentally demonstrate a novel method to mechanically-induce long-period fiber gratings employing laminated plates. For this, three laminated plates were home manufactured with a period around 480 ± 20 µm and a selectable duty cycle. In contrast to conventional MI-LPFGs, the experimental results show that MI-LPFG implemented with the proposed method displays a leading rejection band, and shallow rejection bands in the spectral range from 1100 to 1700 nm. The leading rejection bands in the MI-LPFGs show an attenuation depth greater than 15 dB, and their average background loss can be lower than 0.3 dB. Meanwhile, shallow rejection bands do not reach more than 2 dB. Additionally, the proposed laminated plates offer a simple continuous selectable duty cycle from 10–50%. Laminated plates with the lowest duty cycle enable the inscription of MI-LPFGs with the minimum uniaxial pressure being reported. Finally, the proposed MI-LPFGS with a single leading rejection band allows for the potential coarse wavelength division multiplexing systems based on MI-LPFG.

## 2. Materials and Methods

### 2.1. Laminated Plates Construction

**Figure 1:**
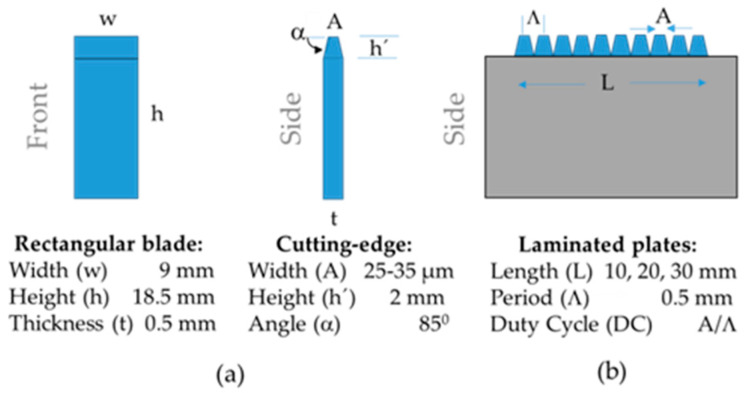
Schematic of: (**a**) rectangular blade, (**b**) laminated plate.

The laminated plate is constructed by piling a group of rectangular blades into a plastic container. The rectangular blades are cut from conventional utility knife blades while using an abrasive disc. Figure 1a illustrates the schematic front and side view of the rectangular blades. They present a single cutting-edge, as can be seen at the top of the side view, where α is the angle of the cutting-edge. Subsequently, the rectangular blades are stacked in a parallel way, and they are placed and fixed by a couple of grub screws in a plastic container, as is also depicted in Figure 1b. Because of the variations in height of the blades and the irregularity of the bottom container is necessary to grid and to polish the top side of the laminated plate to have a flat top side cutting-edge in the rectangular blade assembly. The cutting, assembling, grinding, and polish of the rectangular blades were made at home while using hand tools. 

**Figure 2:**
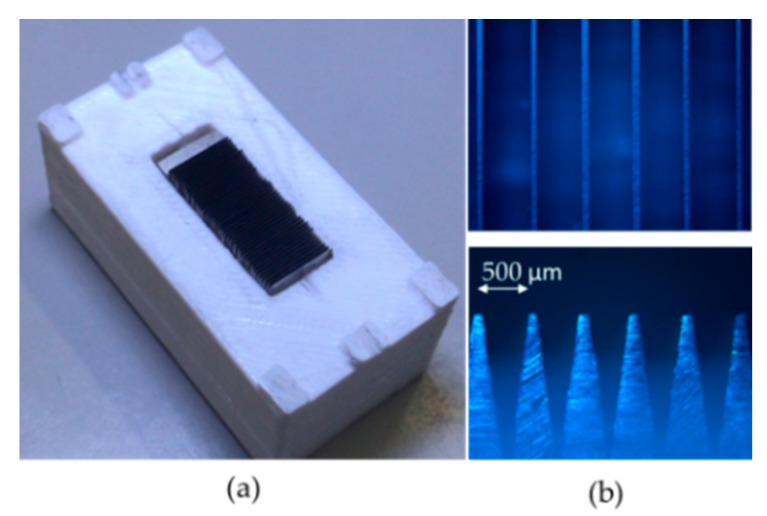
(**a**) Laminated plate construction, (**b**) top and lateral view (4X).

The plastic receptacle was designed by commercial software and reproduced in Polylactic Acid (PLA) by an economical desktop three-dimensional (3D) printer. Once the rectangular blades are prepared and fixed in the plastic container, then the grinding top side of the laminated plate was performed using 350 grain abrasive paper on a hard smooth base. In the polishing process, it was used a decreasing grain polish paper size 500, 1000, and 1500 on a soft smooth base, respectively. Figure 2a shows a constructed laminated plate with 20 mm of length. While the top and bottom pictures of Figure 2b displays the top and lateral view of the mechanical profile at the laminated plate. As can be noted, the period (Λ) and the A parameter vary slightly along with the arrangement of rectangular blades. Besides, the wear of the top laminate plate assemble through the grinding and the polished process offers a simple way to select the A width and the duty cycle.

### 2.2. Refractive Index Modulation by the Laminated Plate

The effective refractive index modulation that is achieved through the proposed MI-LPFG can be described by a transversal and longitudinal refractive index modulation. The transversal refractive index modulation induced in the optical fiber when it is subjected to a uniaxial pressure has been studied while using different methods [31,32]. In this case, the refractive index modulation in the principal axes when a uniaxial pressure is applied along the y-axis can be expressed by [32],

(1) $$n_{x}=n_{x0}+C_{1}\sigma_{x}+C_{2}\sigma_{y}$$

(2) $$n_{y}=n_{y0}+C_{2}\sigma_{x}+C_{1}\sigma_{y}$$

where $n_{x0}$ and $n_{y0}$ are the refractive index observed in the horizontal (x) and vertical (y) transversal axes without uniaxial pressure. $C_{1}$ and $C_{2}$ are the stress-optical coefficients for fused silica. Meanwhile, $\sigma_{x}$ and $\sigma_{y}$ are the x and y stress distribution in [g/mm^2^], respectively, when a uniaxial pressure P_0_ [g/mm] is applied in the vertical axis of the optical fiber, which are defined by [32],

(3) $$\sigma_{x}=\frac{P_{0}}{\pi b}\left[1-\frac{4b^{4}x^{2}}{{\left(b^{2}+x^{2}\right)}^{2}}\right]$$

(4) $$\sigma_{y}=\frac{P_{0}}{\pi b}\left[\frac{4b^{4}}{{\left(b^{2}+x^{2}\right)}^{2}}\right]$$

here, b denotes the outer radius of the optical fiber and x is the coordinate on the horizontal transverse axis of optical fiber. Furthermore, the birefringence (B) by the uniaxial pressure applied in the y-axis, is given by

(5) $$B=n_{x}-n_{y}=\left(n_{x0}-n_{y0}\right)+\left(C_{1}-C_{2}\right)\left(\sigma_{x}-\sigma_{y}\right),$$

where the first term is the residual birefringence of the optical fiber, and the last term is the birefringence component generated by the laminated plate [32]. 

On the other hand, according to the mechanical axial profile of the laminated plate and assuming a very weak cross-sensitivity between transversal and longitudinal strain for small applied perturbation over the fiber [33]. The longitudinal index modulation that is induced by the laminated plate for the i axis can be approached to a periodic rectangular function, given by,

(6) $$n_{i}\left(z\right)=n_{i0}+\frac{A}{\Lambda }\delta n_{i0}\left[1+2\overset{\infty }{\underset{n=1}{\sum }}\sin c\left(\frac{n\pi A}{\Lambda }\right)\cos\left(\frac{n\pi z}{\Lambda }\right)\right]\;\mathrm{for}\;z\leq \left|\frac{L}{2}\right|$$

**Figure 3:**
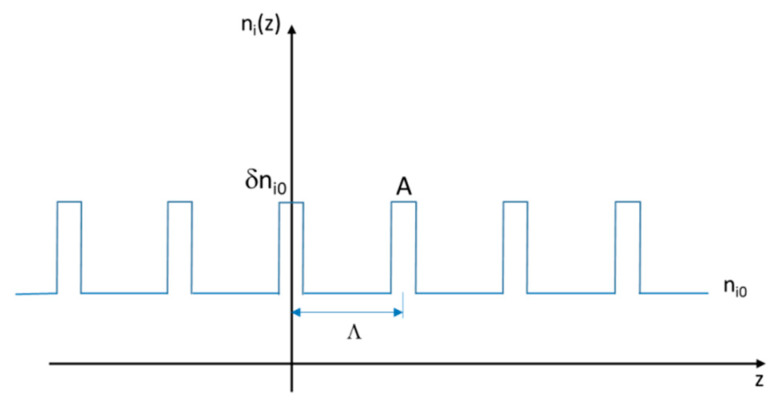
Longitudinal refractive index modulation for the i (x,y) axis.

The aforementioned $n_{i0}$ is the i axis core refractive index, ${\delta n}_{i0}$ is the refractive index perturbation generated by the laminated plate, and L is the length of the laminated plate. The Figure 3 illustrates the longitudinal index modulation, where A is the top width of the perturbation ${\delta n}_{0i}$ and Λ is the modulation period.

### 2.3. Experimental Setup

**Figure 4:**
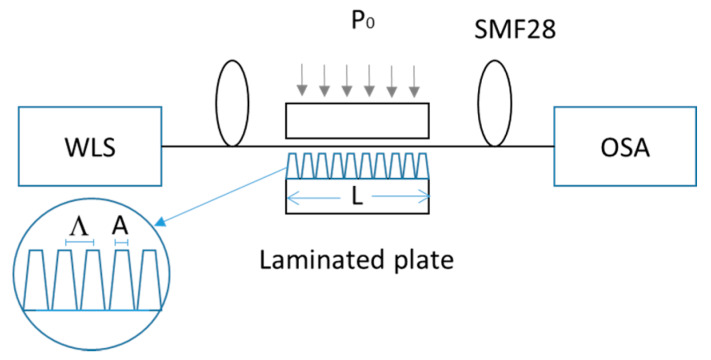
Experimental configuration.

Three laminated plates: L10, L20, and L30 with lengths of 10, 20, and 30 mm were prepared following the methodology that was described in the previous section. Table 1 shows the main sizes of these plates. Where, $\bar{\Lambda }$, $\bar{A}$ are their average values of the Λ and A, while $\sigma_{\Lambda }$, $\sigma_{A}$ are their corresponding standard deviations. The estimated error in the measurement of Λ and A was 10 µm. The average duty cycle (ADC) is defined by $\mathrm{ADC}=\frac{\bar{A}}{\bar{\Lambda }}\left(100\%\right)$ and it was calculated for each laminated plate. Figure 4 illustrates the schematic of the experimental setup employed for the spectrum transmission characterization of the laminated plates on single-mode optical fiber (SMF28). The experimental setup consists of a white light source (WLS: AQ-4303B) at the input and a spectral optical analyzer (OSA: AQ-6315A) in the output, which are optically communicated by the SMF28. The single-mode optical fiber is placed on the press rig, as shown in Figure 4. The long-period grating is mechanically-induced by pressing the single-mode optical fiber over the laminated plate through the top flat plate. Adjusting the static weight that was applied on the top flat plate was utilized to regulate the uniaxial pressure over the optical fiber. In the implementation of the MI-LPFGs, the optical fiber always kept the protective plastic coating. 

## 3. Results

**Figure 5:**
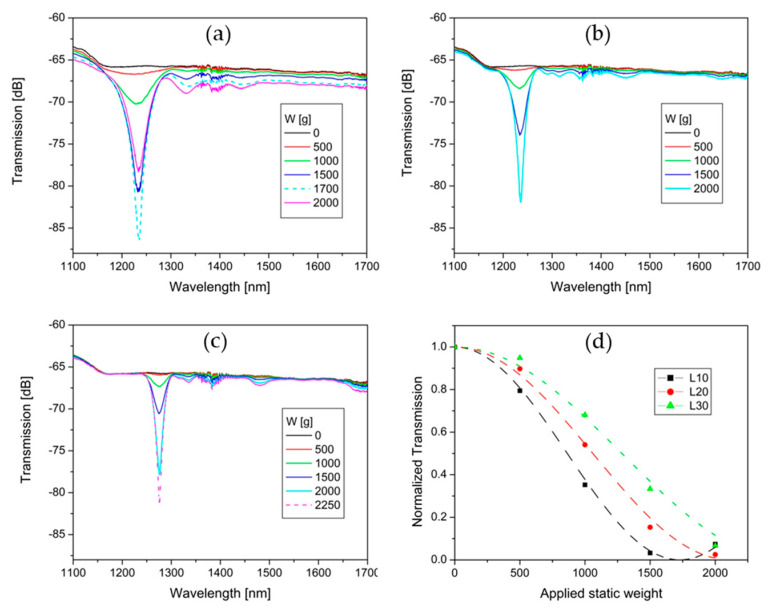
Spectrum transmission evolution for (**a**) L10, (**b**) L20, and (**c**) L30 under the increase of applied static weight. (**d**) Attenuation depth of the leading rejection bands versus applied static weight.

Figure 5 illustrates the spectrum transmission evolution of L10, L20, and L30 under static weight (W) applied by steps of 500 g. In Figure 5a, L10 presents a leading rejection band at 1234 nm with three lateral lobes on the right. As can be seen, the attenuation depth that corresponds to 2000 g is in the overcoupling region of the MI-LPFG. Additionally, the spectrum transmission related to the static weight of 1700 g has been added to show the maximum attenuation depth of the leading rejection band (20 dB). Meanwhile, the average background loss grew when the applied static load was increased. In this case, the maximum average background loss is around 1.8 dB for 1700 g of weight. On the other hand, L20 presents a leading rejection band at 1237 nm, which achieves a maximum of 16 dB for 2000 g; see Figure 5b. The leading rejection band presents two lateral lobes on the right. Additionally, the spectrum transmission presents two shallow rejection bands at 1450 nm and 1645 nm. In this case, there is no overcoupling observed. The average background loss for the maximum attenuation (15 dB) of the leading rejection band is lower than 0.3 dB. 

In the same way, L30 presents a leading rejection band at 1276 nm with two lateral lobes in the right and two shallow rejection bands at 1480 nm and 1690 nm; see Figure 5c. The average background loss for the maximum attenuation depth (15.5 dB) of the leading rejection band is lower than 0.2 dB. The spectrum transmission that corresponds to the static weight of 2250 g has been added to show that the attenuation depth of the leading rejection band can be deeper than 15 dB. Figure 5d shows the normalized transmission of the leading rejection bands (symbols) versus the increase of the applied static weight corresponding to the experimental results from Figure 5a–c. According to Starodubov et al. [34], the colored dashed curves correspond to the function $T\left(W\right)=\cos^{2}\left(D\cdot L/2\right)$ that best fits each colored symbol. Where D is the coupling coefficient, L is the length of the LPFG, and W is the applied static weight. D is proportional to the overlap integral of the core and cladding modes and the index modulation of the grating. In turn, the index modulation depends on W. In this way, we can find a relationship between the coupling coefficient D and W, as follows: D=factor·W. For a first approach, D=0.018·W for L10, while D=0.007·W for L20, and D=0.004·W for L30. The described MI-LPFGs showed good stability and high repeatability at room temperature and uniaxial pressure lower than 3000 g/mm. The uncertainty of the central resonant wavelengths for the leading rejection band of L10, L20, and L30 was ±2 nm for the above conditions.

**Figure 6:**
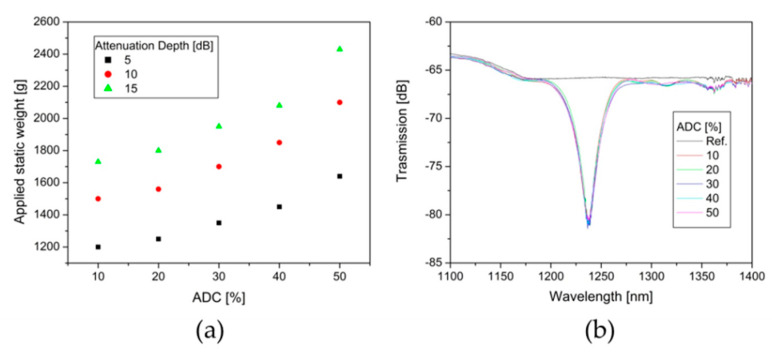
(**a**) Static weights grow for attenuation depth of 5, 10, and 15 dB in the leading rejection band as a function of average duty cycle (ADC). (**b**) Transmission spectrum evolution of the leading rejection band with 15 dB for different values of ADC.

A new laminated plate (NL20) was prepared following the grinding and polished process before described in order to understand the impact of the ADC on the spectrum transmission in the MI-LPFGs. By this process the ADC was varied from 10 to 50% in steps of around 10%. Subsequently, the uniaxial pressure on the optical fiber over the laminated plate was controlled by the static weight applied. Figure 6a shows the evolution of the static weight required to reach attenuation depths of 5, 10, and 15 dB in the leading rejection band when the ADC increases from 10 to 50%. As expected, the static weight required to reach the defined attenuation depths grows in a nonlinear way as the ADC increase. Figure 6b depicts the spectrum transmission of the leading rejection band with attenuation depth of 15 dB obtained for each one of the different ADC in the NL20. As can be observed, the spectrum transmission practically does not present essential changes regarding bandwidth and background loss. Additionally, not additional attenuation bands or a significant increase of the attenuation depth in the shallow rejection bands were observed. We observed similar behavior for the spectrum transmission of the leading rejection band for attenuation depths of 5 and 10 dB.

## 4. Analysis of Results

As is well-known, the spectrum transmission of conventional MI-LPFGs is characterized by multiple rejection bands with deeper attenuation bands for high order couplings. In contrast, the proposed laminated plates allow for MI-LPFGs with only a stable leading rejection band and lower secondary shallow attenuations bands for high order couplings. Where, the attenuation depth of the secondary attenuation bands are more than one order lower with respect to the attenuation depth of the leading rejection bands, as can be observed in Figure 5a–c. From these results, we infer that the generation of a leading rejection band is due to the longitudinal rectangular index modulation with a high raise-space and small variation of ${\delta n}_{i0}$ along the MI-LPFGs. These properties promote a strong coupling between the fundamental mode in the core to a single high-order cladding mode and a weak coupling to lower and higher order cladding modes. To the best of our knowledge, this is the first time that MI-LPFGs with a single leading rejection band have been reported.

The leading rejection bands present other attractive optical and mechanical characteristics with respect to rejection bands in conventional MI-LPFGs. The leading rejection bands are located below 1300 nm with a contrast >15 dB for a 480 µm period, see Figure 5a–c. Meanwhile, conventional MI-LPFGs with a similar period present high contrast rejection bands above 1400 nm. Additionally, it can be noted in Figure 5 that the wavelength band from 1500 to 1600 nm is free of attenuation peaks, and the average background loss is lower than 0.2 dB for L20 and L30 cases. These properties show the potential coarse wavelength division multiplexing of sensors or devices based in MI-LPFG in the bands at 1300 nm and 1500 nm. Additionally, the leading rejection band in L10 shows an attenuation depth of 20 dB for 1700 g and 10 mm of length, see Figure 5a. To the best of our knowledge, these are the minimum static weight, and grating length reported to obtain a rejection band with 20 dB of attenuation depth in MI-LPFGs. Furthermore, a low uniaxial pressure noticeably reduces the probability of failure in the optical fiber. In this sense, lower uniaxial pressures can be used to inscribe MI-LPFGs by laminated plates, where the results are of great interest in distinct applications [16,17,18,19,20,21,22,23,24,25,26]. On the other hand, the normalized transmission of the leading rejection bands shows similar behavior with respect to the rejection bands in conventional LPFGs, as depicted in Figure 5d, where one can find good correlation between the experimental results and the theory.

Figure 6a shows an increase of the static weight that is required to obtain the 15 dB of attenuation depth in the leading rejection band when the ADC grow from 10% to 50%. This behavior can be expected if we consider that a lower ADC implies a larger uniaxial pressure over the optical fiber for the same static weight applied. The similar behavior of the static weight required for 5 and 10 dB of attenuation depth was observed; see Figure 6a. Besides, we found experimentally that the duty cycle has an insignificant influence on the spectrum transmission of the leading rejection band for 15 dB, as shown in Figure 6b. There is no notable change in the bandwidth, and there is not a wavelength shift of the central resonant wavelength for the different values of ADC. Additionally, we did not observe the mode coupling to high order diffraction modes for low values of ADC, as other methods reported [35]. The spectrum transmission of the leading rejection band for attenuation depths of 5 and 10 dB presents a similar behavior.

The spectrum transmission of MI-LPFGs depends on the laminated plate quality. This, in turn, depends on the precision of the blade sizes and cutting-edge variation. In this case, the cutting-edge diameter of the blades varies from 25–35 µm. Additionally, the cutting-edge of the blades do not maintain their vertical symmetry, and the cutting-edge angle varies ±3°. On the other hand, the quality of the laminated plate also depends on the fabrication process. The manual cutting of the rectangular blades and the hand assembling process produced a significant scattering of the period and the A diameter, as can be seen in Table 1. Despite these drawbacks, the proposed laminated plates are attractive for practical applications. Further improvements on the laminated plates can be expected by using high-quality blades and a proper assembling process in a mechanical workshop.

## 5. Conclusions

In this work, a new method for implementing MI-LPFGs using laminated plates is presented. According to the experimental results, the proposed MI-LPFGs exhibit a spectrum transmission with a leading rejection band, and secondary shallow attenuation bands one order smaller. The leading rejection bands stand out for their high attenuation depth and low average background losses. To our best knowledge, this is the first time that MI-LPFGS with a single rejection band has been reported. Additionally, we experimentally analyze the influence of the ADC on the leading rejection band in the MI-LPFGs. In this respect, we report a MI-LPFG with an ADC of 20%, 10 mm of length, and 1700 g of static weight applied to obtain a leading rejection band with 20 dB of attenuation depth. As far as we know, the proposed laminated plates with low ADC require the lowest uniaxial pressure being reported on MI-LPFGs. Furthermore, the concept of LPFGs with a leading rejection band can be extended to other inscription methods, like UV or the fs-lasers. Finally, the proposed MI-LPFGs show the potential implementation of wavelength division multiplexing of sensors or devices based on MI-LPFGs at 1300 nm and 1500 nm bands for the first time.

## Figures and Tables

**Table 1:** Laminated plates constructed: Length and average values.

Laminated Plate	L [mm]	$\bar{\Lambda }$	$\bar{A}$	$\sigma_{\Lambda }[µm]$	$\sigma_{A}[µm]$	ADC [%]
L10	10	460	94	20	15	20.4
L20	20	470	92	18	7	19.6
L30	30	490	98	24	16	20.0
